# Predictors and clinical impact of intraoperative coronary sinus triggers in patients with paroxysmal atrial fibrillation undergoing radiofrequency catheter ablation

**DOI:** 10.3389/fcvm.2026.1771174

**Published:** 2026-03-26

**Authors:** Nishant Yadav, Yan Dong, Qiushi Chen, Li Jiang, Yuan He, Fengxiang Zhang

**Affiliations:** Section of Pacing and Electrophysiology, Department of Cardiology, The First affiliated Hospital of Nanjing Medical University, Nanjing, China

**Keywords:** atrial fibrillation, catheter ablation, coronary sinus trigger, CPVI, recurrence

## Abstract

**Aim:**

This study aims to investigate the incidence and clinical impact of intraoperative coronary sinus (CS) triggers in patients with paroxysmal atrial fibrillation (PAF) undergoing radiofrequency catheter ablation.

**Methods:**

Patients with symptomatic drug-refractory PAF who underwent index catheter ablation were enrolled. Patients were assigned into either the CS trigger group or pulmonary vein (PV) trigger group based on the results of electrophysiological studies of induced AF trigger. Any documented atrial tachyarrhythmia lasting ≥30 s after a 3-month blanking period, without any anti-arrhythmic drugs during post-ablation follow-up was defined as recurrence.

**Results:**

The incidence of CS triggers among patients undergoing index ablation for PAF was 5.3%. Patients in the CS trigger group were younger (53.0 ± 11.8 vs. 60.5 ± 9.0 years; *P* < 0.01) and had a smaller left atrial diameter (LAD) (36.8 ± 4.0 vs. 38.7 ± 3.8 mm; *P* = 0.05) than those in the PV trigger group. Age [odds ratio [OR] 0.93, 95% confidence interval [CI] 0.89–0.98; *P* < 0.01] and LAD (OR 0.88, 95% CI 0.77–1.00; *P* = 0.05) were identified as independent predictors of CS triggers. Over a mean follow-up period of 13.7 ± 8.3 months, there was no significant difference in freedom from atrial tachyarrhythmias between the CS trigger and the PV trigger groups (76.5% vs. 84.2%; log-rank *P* = 0.50).

**Conclusion:**

CS triggers are most commonly observed in relatively younger patients with a smaller LAD. The presence of a CS trigger does not adversely affect post-ablation rhythm outcomes when CS trigger ablation is incorporated into PAF management.

## Introduction

Atrial fibrillation (AF) is one of the most frequently encountered tachyarrhythmias. It not only substantially reduces exercise tolerance and quality of life but also significantly adds to the risk of heart failure, stroke, and mortality (1, 2). Currently, circumferential pulmonary vein isolation (PVI) serves as the mainstay of catheter ablation for AF, and its safety and efficacy have been well established (3–5). However, accumulating evidence indicates that non-pulmonary vein (non-PV) triggers play an important role in the initiation and maintenance of AF. The non-PV triggers are also significantly associated with post-PVI recurrence in patients with paroxysmal atrial fibrillation (PAF) (6). Non-PV triggers mainly include the superior vena cava (SVC), posterior free wall of the left atrium, coronary sinus (CS), ligament of Marshall (LOM), among others. The incidence of non-PV triggers in PAF patients ranges from 28.0% to 31.5% (7, 8).

Previous studies have shown anatomical associations between the left atrium and the CS musculature. Anatomical and electrical connections between the left atrium and CS muscle bundles are critical for the initiation and maintenance of AF (9, 10). It has also been shown that CS and left atrial frequency-dependent unidirectional block are linked to AF induction (9). However, existing evidence remains inconsistent, highlighting the need to better characterize CS triggers and evaluate their clinical impact. This study aims to evaluate the intraprocedural incidence of CS triggers and their effect on the post-ablation success rate in PAF patients.

## Methods

### Study population

**Figure 1:**
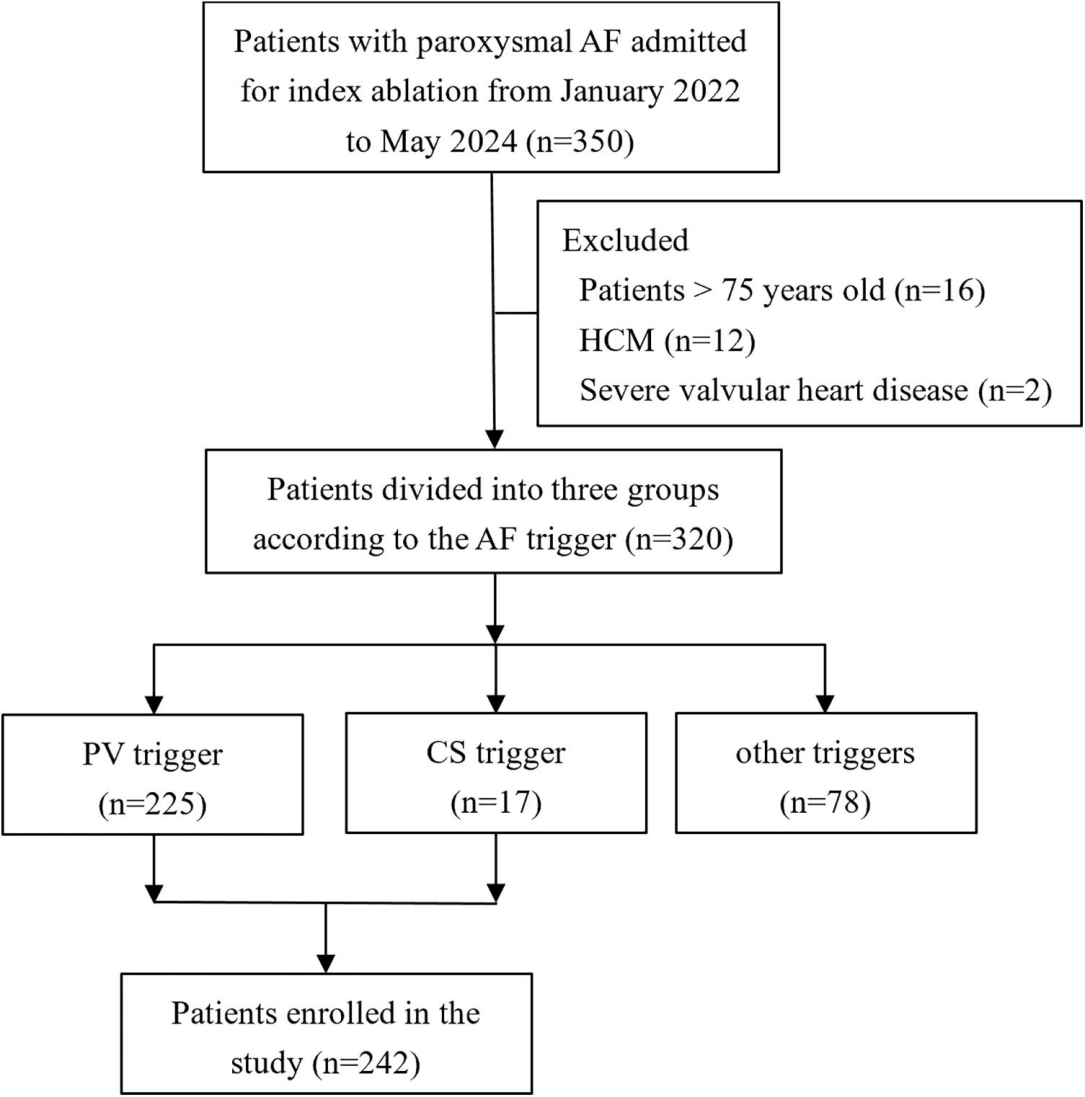
The flow chart of the study. AF, atrial fibrillation; HCM, hypertrophic cardiomyopathy; PV, pulmonary vein; CS, coronary sinus.

We consecutively enrolled patients with symptomatic PAF admitted for index radiofrequency catheter ablation in the First Affiliated Hospital of Nanjing Medical University from January 2022 to May 2024. Initially, 350 patients were screened. Patients with age >75 years old (*n* = 16), hypertrophic cardiomyopathy (*n* = 12), or severe valvular heart disease (*n* = 2) were excluded. The remaining 320 patients were then divided into three groups according to the identified AF trigger: PV trigger (*n* = 225), CS trigger (*n* = 17), and other triggers (*n* = 78). Patients with PV trigger or CS trigger were finally enrolled in this study, thus a total of 242 patients included in the analysis. Figure 1 shows the flow chart of this study. This study was conducted in compliance with the Declaration of Helsinki and was approved by the Ethics Review Committee of the First Affiliated Hospital of Nanjing Medical University (2024-SR-669).

### Perioperative management and mapping

All anti-arrhythmic drugs except amiodarone were stopped for at least five half-lives, and uninterrupted oral anticoagulants were administered for at least 3 weeks prior to the procedure. Transesophageal echocardiography (TEE) or cardiac computed tomography (CT) was performed ≤48 h prior to the ablation to rule out thrombus in the left atrial appendage. The procedure was performed with the patient under conscious sedation with infusion of intravenous fentanyl. Three-dimensional mapping system (CARTO 3, Biosense Webster, Diamond Bar, CA, USA) was used to guide the procedure. A multipolar catheter (PentaRay; Biosense Webster, Diamond Bar, CA, USA) was used to map the pulmonary vein ostia, and a 3.5 mm irrigated-tip ablation catheter (Thermocool Smarttouch/Thermocool Smarttouch Surround Flow, Biosense Webster, Diamond Bar, CA, USA) was used for ablation. Intravenous heparin was titrated throughout the procedure to maintain the activated clotting time (ACT) in the range of 300–350 s.

### Electrophysiological studies for induction of AF triggers

A uniform induction protocol was implemented in all the enrolled patients, prior to performing PVI, under sinus rhythm. A decapolar catheter was positioned in the CS, two quadripolar catheters were placed in superior vena cava (SVC) and right ventricular apex (RVA) respectively, PentaRay mapping catheter was positioned in right superior pulmonary vein (RSPV), with the ablation catheter in left superior pulmonary vein (LSPV). Then, in baseline state, high frequency stimulation (200 ms, 30 s) was applied to the CS proximal, distal, SVC, RSPV and LSPV respectively, until AF was induced. If AF could not be induced after the completion of above provocation protocol, isoproterenol (ISO) (4 μg/min) was administered intravenously until the heart rate increased more than 20% compared with the baseline, and the above provocation protocol was repeated until AF was induced. If AF could still not be induced after the completion of above provocation protocols, adenosine triphosphate (ATP) 40 mg was administered intravenously under RVA pacing. If AF/atrial tachycardia (AT) still did not occur, high frequency stimulation was applied again as mentioned above until AF/AT occurred. If AF remained non-inducible after completion of the provocation protocol, AF was designated as non-inducible. AF inducibility is defined as any episode of AF provoked by non-catheter contact factors during the induction protocol and lasting for ≥30 s. The first atrial beat triggering AF is regarded as the trigger activity and used to identify the trigger focus (13). A CS trigger is defined as the earliest atrial activation recorded by the decapolar catheter positioned within the CS, preceding other atrial electrograms, and reproducibly initiating AF during the induction protocol. Ablation of the CS trigger involves targeting the earliest activation site, initially within the left atrial chamber at the LA-CS adjunction, followed by ablation at the corresponding area within the lumen of the CS, based on precise mapping and localization (14). The procedural endpoint for CS trigger ablation was complete elimination of the earliest ectopic activity originating from the CS and non-inducibility of AF after using the same standardized induction protocol applied before ablation. Ablation within the CS was performed using irrigated radiofrequency energy at 25–30 W, temperature limited to 43°C, with continuous impedance monitoring and careful power titration to avoid CS injury.

### Circumferential pulmonary vein isolation (PVI)

The sites for radiofrequency ablation lesions were selected approximately 1 cm beyond the ostia of the pulmonary vein to ensure antral isolation (15). PVI was performed in power-controlled mode with 30–50 W, contact force 10–20 g with a temperature limit of 43 °C. Lesions were guided by the ablation index (AI). Target AI values were 500 at the anterior wall, 450–500 at the posterior-superior wall, and 350–400 at the posterior-inferior wall, with point-by-point ablation until complete PV isolation was achieved. The endpoint of PVI was determined to be the bidirectional conduction block between the PVs and LA after 30 min observation time post-isolation, and intravenous infusion of isoproterenol and ATP. Direct current cardioversion was performed to convert AF into sinus rhythm if AF still persisted. Ablation targeting the focal or critical isthmus was performed in case of spontaneous atrial flutter (AFL)/AT during the ablation.

### Post-ablation management and follow-up

Any documented atrial tachyarrhythmia lasting ≥30 s after a 3-month blanking period, without any anti-arrhythmic drugs during post-ablation follow-up was defined as recurrence. Patients remained on anticoagulation therapy for at least 3 months post-ablation. The antiarrhythmic drug (AAD) was given only for 3 months post-ablation. All patients received a proton pump inhibitor for 6 weeks post-ablation.

The 24-hour Holter ECG was recorded at 1st, 3rd and 6th month post-ablation, and a 7-day Holter ECG was recorded at 12th month post-ablation. The patient was advised for hospital visit as soon as palpitation and/or other discomforts occurred, so that ECG could be done to determine whether AF recurred. Procedure related complications/ adverse events which were looked for were sinus node injury, phrenic nerve injury, cardiac tamponade/effusion, stroke/systemic embolism, atrium-esophageal fistula, pulmonary vein stenosis, and mortality.

### Statistical analysis

Continuous variables following a normal distribution were expressed as mean ± standard deviation, with comparisons performed via the two-sample *t*-test. For non-normally distributed continuous variables, they were reported as median (interquartile range), and their differences were assessed by the Mann–Whitney *U*-test. Categorical variables were summarized as frequency (percentage) and analysed with the *χ*² test or Fisher's exact test. Logistic regression analyses were performed to identify independent predictors of CS triggers. Kaplan–Meier survival curves were used to display the freedom from atrial tachyarrhythmias survival rate, and compared using the log-rank test. A two-sided *P*-value <0.05 was considered statistically significant.

## Results

### Patient characteristics

Of the 320 patients with symptomatic drug-refractory PAF undergoing index catheter ablation, the incidence of CS triggers was 5.3% (17/320). After excluding 78 patients with other AF triggers, the remaining 242 were divided into two target groups: CS trigger (*n* = 17) and PV trigger (*n* = 225). The mean age of the CS trigger group was significantly lower than that of the PV trigger group (53.0 ± 11.8 vs. 60.5 ± 9.0 years; *P* < 0.01). Although a higher proportion of females were observed in the CS trigger group (58.8% vs. 36.4%), this difference was not statistically significant (*P* = 0.12). Left atrial diameter (LAD) was lower in the CS trigger group as compared to the PV trigger group (36.8 ± 4.0 mm vs. 38.7 ± 3.8 mm; *P* = 0.05). The CHA₂DS₂-VASc score was comparable between the two groups [CS trigger: 1 [0–2] vs. PV trigger: 1 [1–3]; *P* = 0.14]. In contrast, the HAS-BLED score was significantly lower in the CS trigger group [1 (0–2)] than that in the PV trigger group [1 (1–2); *P* = 0.04]. The baseline demographic characteristics of the patients are summarized in Table 1.

**Table 1:** Baseline characteristics.

Parameters	CS trigger (*n* = 17)	PV trigger (*n* = 225)	*P* value
Female (%)	10 (58.8)	82 (36.4)	0.12
Age (year)	53.0 ± 11.8	60.5 ± 9.0	<0.01
BMI (kg/m^2^)	25.3 ± 2.7	25.1 ± 3.0	0.77
Smoking (%)	2 (11.8)	59 (26.2)	0.30
Drinking (%)	2 (11.8)	38 (16.9)	0.83
Duration (month)	12.0 (12.0, 36.0)	12.0 (3.0, 36.0)	0.3
CHA_2_DS_2_-VASc score	1.0 (0.0, 2.0)	1.0 (1.0, 3.0)	0.14
HAS-BLED score	1.0 (0.0, 2.0)	2.0 (1.0, 2.0)	0.04
Comorbidities (%)			
Ischemic stroke	0	22 (9.8)	0.36
Coronary heart disease	0	35 (15.6)	0.16
Hypertension	6 (35.3)	114 (50.7)	0.33
Diabetes mellitus	0	38 (16.9)	0.13
Heart failure	0	4 (1.8)	1.00
Medication use (%)			
Amiodarone	1 (5.9)	8 (3.6)	1.00
Propafenone	1 (5.9)	14 (6.2)	1.00
*β*-blocker	3 (17.6)	79 (35.1)	0.23
CCB	2 (11.8)	47 (20.9)	0.56
ACEI/ARB	3 (17.6)	49 (21.8)	0.93
Diuretic	1 (5.9)	13 (5.8)	1.00
statin	3 (17.6)	83 (36.9)	0.18
LAD (mm)	36.8 ± 4.0	38.7 ± 3.8	0.05
RAD (mm)	34.4 ± 3.1	35.4 ± 4.1	0.31
LVDD (mm)	48.0 ± 2.6	47.6 ± 4.0	0.68
LVDS (mm)	31.4 ± 2.0	31.8 ± 3.3	0.61
LVEF (%)	63.7 ± 2.7	62.8 ± 3.6	0.33

BMI, body mass index; CCB, calcium channel blocker; ACEI, angiotensin converting enzyme inhibitor; ARB, angiotensin receptor blocker; LAD, left atrium diameter; RAD, right atrium diameter; LVDD, left ventricular end-diastolic diameter; LVDS, left ventricular end-systolic diameter; LVEF, left ventricular ejection fraction; CS, coronary sinus; PV, pulmonary vein.

### Procedural characteristics and perioperative complications

Procedural characteristics and perioperative complications are presented in Table 2. The CS trigger group had a longer, but not statistically significant, procedure time compared to the PV trigger group (182.4 ± 22.8 vs. 168.9 ± 37.2 min; *P* = 0.14). The proportion of the mitral isthmus (MI) ablation was significantly higher in the CS trigger group (11.8% vs. 0%; *P* < 0.01). No other significant differences in ablation strategies were observed. The overall incidence of perioperative complications was low and comparable between groups (CS trigger group 0% vs. PV trigger group 1.8%, *P* = 1.00). The PV trigger group consist of one case (0.4%) of pericardial effusion, one case (0.4%) of thromboembolic event, and two cases (0.9%) of vascular complications. No phrenic nerve injury, sinus node injury, or mortality occurred in either group.

**Table 2:** Procedural characteristics.

Parameters	CS trigger (*n* = 17)	PV trigger (*n* = 225)	*P* value
Procedure time (min)	182.4 ± 22.8	168.9 ± 37.2	0.14
Fluoroscopy time (min)	6.4 ± 2.3	8.2 ± 5.8	0.20
Left atrial low-voltage (%)	0	5 (2.2)	1.00
Roof line (%)	4 (23.5)	84 (37.3)	0.38
Mitral isthmus ablation (%)	2 (11.8)	0	<0.01
Cavotricuspid isthmus ablation (%)	4 (23.5)	44 (19.6)	0.94
SVCI (%)	17 (100.0)	198 (88.0)	0.26
Perioperative complications (%)	0	4 (1.8)	1.00
Pericardial effusion	0	1 (0.4)	1.00
Thromboembolic events	0	1 (0.4)	1.00
Vascular complications	0	2 (0.9)	1.00
Phrenic nerve injury	0	0	–
Sinus node injury	0	0	–
Death	0	0	–

SVCI, superior vena cava isolation; CS, coronary sinus; PV, pulmonary vein.

### Predictors of CS trigger

**Figure 2:**
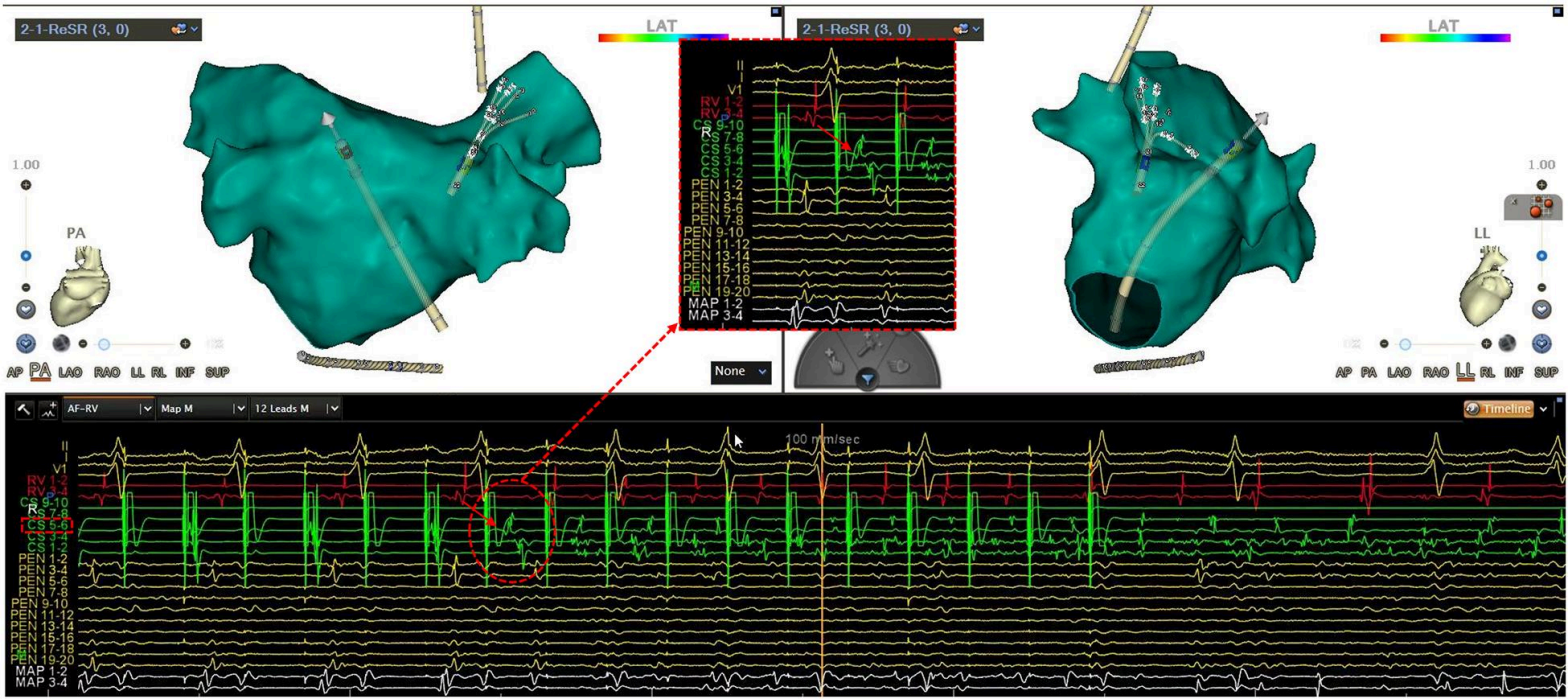
An example of CS56-triggered AF in one patient with PAF. CS, coronary sinus; PAF, paroxysmal atrial fibrillation. With high-frequency stimulation (200 ms, 30 s) during isoproterenol infusion, the earliest atrial activation was observed at CS 5–6 (arrow), reproducibly initiating AF.

Logistic regression analysis for the predictors of CS triggers was analysed (Table 3). In the univariable model, age [odds ratio [OR] 0.93, 95% confidence interval [CI] 0.89–0.98; *P* < 0.01] and LAD (OR 0.88, 95% CI 0.77–1.00; *P* = 0.05) were identified as independent predictor of CS trigger. The multivariable analysis was not done given the low number of events. Figure 2 shows the example of CS56-triggered AF in one of the cases in this cohort.

**Table 3:** Logistic regression analyses to identify predictors of CS trigger.

Parameters	Univariable analysis	*P* value
	OR (95% CI)	
Female (%)	2.49 (0.92–7.1)	0.07
Age (year)	0.93 (0.89–0.98)	<0.01
BMI (kg/m^2^)	1.02 (0.87–1.21)	0.77
Smoking (%)	0.38 (0.06–1.38)	0.20
Drinking (%)	0.66 (0.1–2.46)	0.59
β-blocker (%)	0.4 (0.09–1.26)	0.16
Hypertension	0.53 (0.18–1.45)	0.23
LAD (mm)	0.88 (0.77–1.00)	0.05
RAD (mm)	0.94 (0.82–1.06)	0.31

BMI, body mass index; LAD, left atrium diameter; RAD, right atrium diameter; CS, coronary sinus; OR, odds ratio; CI, confidence interval.

### Efficacy

**Figure 3:**
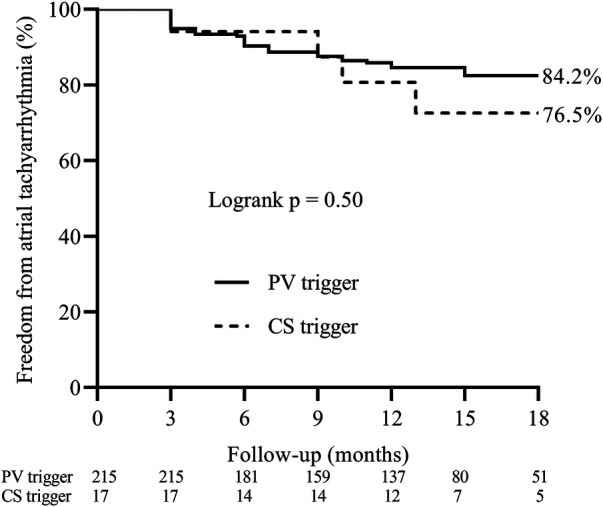
Kaplan–meier curve for freedom from atrial tachyarrhythmias after the index ablation.

Figure 3 presents the Kaplan–Meier analysis for evaluating recurrence-free survival. Over a mean follow-up period of 13.7 ± 8.3 months, 10 patients were lost to follow-up, resulting in 232 patients who completed the study. No significant difference was observed between the CS trigger group and the PV trigger group (76.5% vs. 84.2%; log-rank *P* = 0.50).

## Discussion

### Main findings

In this study, the incidence of CS triggers in patients undergoing index-ablation for PAF was 5.3% having a higher prevalence in younger patients with a smaller LAD. Although the long-term rhythm outcomes were comparable between the two groups, the electrophysiological characteristics of the CS trigger suggest that this non-PV foci remains clinically significant.

CS triggers are considered important non-PV drivers due to the unique muscular sleeves and complex myocardial connections between the CS and the left atrium (9, 10). Previous literature has reported a CS trigger prevalence ranging in between 5% and 10% in AF ablation cohorts (13, 16), making our observed incidence comparable. In this study, the incidence of CS triggers shows a higher percentage in younger and female patients. This demographic trend is consistent with the several previous reports suggesting age-and sex-related differences in non-PV triggers (8, 17, 18). Although the limited number of events in our cohort precluded a stable multivariable analysis, the univariate association of female sex with CS triggers remains clinically noteworthy. Watanabe et al. (8) and Oraii et al. (17) reported that female sex was a significant risk factor associated with non-PV triggers during index ablation for AF. In addition, patients with CS triggers in our study exhibited slightly smaller LAD as compared to those with PV triggers. This may represent a phenotype with less advanced structural remodeling, in whom focal non-PV triggers remain dominant drivers of AF. It is important to recognize that in our study population, female sex, younger age, and smaller LAD often clustered together. These interrelated factors suggest a specific patient profile where the CS musculature acts as a dominant driver. Regarding procedural factors, although the proportion of MI ablation differed between the groups, MI ablation was excluded from the regression analysis, because it occurred only after trigger identification and therefore could not serve as a baseline predictor of CS triggers.

The shared anatomical substrate between the CS and left atrium—especially the presence of slow conduction zones and rate-dependent block—may facilitate arrhythmogenic wavefront propagation (9, 10). The previous studies have shown that targeted CS ablation yielded high success rates without increasing complications (11, 12). In a cohort study by Haïssaguerre et al. (11), among the 45 patients (15 PAF and 30 PsAF) included in the study, it was shown that the fibrillatory cycle length was significantly prolonged and the AF persisting after PV isolation was terminated in 35% of patients by CS region targeted ablation. At 10 ± 3 months of follow-up, 80% of the PAF patients were arrhythmia-free. In another randomized controlled trial by Kuo et al. (12), 35 patients with AF undergoing index ablation were randomized to the standard ablation or standard plus elimination of CS-LA connection group, it was shown that the additional elimination of CS-LA connection decreased the percentage of recurrences.

However, rhythm outcomes in our CS-trigger cohort did not differ significantly from the PV-trigger group. This may be attributed to the fact that focal ablation of the earliest CS activation may not thoroughly eliminate all the CS–LA conduction pathways, especially in patients with multiple interconnections. The undetected or incompletely ablated CS triggers may contribute to recurrence, as highlighted by Lin et al. (7) and Watanabe et al. (8). This underscores the need for systematic and reproducible non-PV trigger induction protocols (19).

Certain earlier investigations suggested a marginally higher recurrence rate in patients with CS triggers, potentially due to incomplete ablation or variability in detection techniques (16, 20). Advances in mapping systems and ablation catheters in recent years, and verification of elimination of LA-CS connection may contribute to improved procedural success rate in the future.

### Limitations

This study has its limitations. First of all, this is a retrospective study in a single-center, having a limited sample size. A prospective, multi-center cohort study needs to be conducted to overcome this limitation and evaluate the outcome of CS trigger ablation in larger sample size to establish its efficacy. Second, in this study we used 24 h and 7 days Holter only during the follow-up to monitor the rhythm of the patients, so, we may have missed out on the recurrence of atrial arrhythmia in few patients. Third, left atrial volume index (LAVi) is a more accurate and methodologically superior parameter than linear LAD for assessing atrial size. However, as LAVi was not systematically measured, we are unable to provide this data for this cohort.

## Conclusion

CS triggers are most commonly observed in comparatively younger patients with smaller LAD. The presence of CS trigger does not adversely affect post-ablation rhythm outcomes when CS trigger ablation is incorporated into PAF management.

## Data Availability

The original contributions presented in the study are included in the article/Supplementary Material, further inquiries can be directed to the corresponding author upon reasonable request.
